# Dosimetric Comparison of VMAT Alone and VMAT with HDR Brachytherapy Boost Using Clinical and Biological Dose Models in Localized Prostate Cancer

**DOI:** 10.3390/curroncol32060360

**Published:** 2025-06-19

**Authors:** Manuel Guhlich, Olga Knaus, Arne Strauss, Laura Anna Fischer, Jann Fischer, Stephanie Bendrich, Sandra Donath, Leif Hendrik Dröge, Martin Leu, Stefan Rieken, Annemarie Uhlig, Markus Anton Schirmer, Andrea Hille

**Affiliations:** 1 Clinic of Radiotherapy and Radiation Oncology, University Medical Center Göttingen, Robert-Koch-Str. 40, 37075 Göttingen, Germany; olga.knaus@med.uni-goettingen.de (O.K.); lauraanna.fischer@med.uni-goettingen.de (L.A.F.); jann.fischer@med.uni-goettingen.de (J.F.); stephanie.bendrich@med.uni-goettingen.de (S.B.); sandra.donath@med.uni-goettingen.de (S.D.); hendrik.droege@med.uni-goettingen.de (L.H.D.); martin.leu@med.uni-goettingen.de (M.L.); stefan.rieken@med.uni-goettingen.de (S.R.); mschirmer@med.uni-goettingen.de (M.A.S.); ahille@med.uni-goettingen.de (A.H.); 2 Department of Urology, University Medical Center Göttingen, Robert-Koch-Str. 40, 37075 Göttingen, Germany; astrauss@med.uni-goettingen.de (A.S.); annemarie.uhlig@med.uni-goettingen.de (A.U.)

**Keywords:** prostate cancer, radiotherapy, high-dose-rate brachytherapy (HDR-BT), external beam radiotherapy (EBRT), biologically effective dose (BED), organs at risk (OARs), dose escalation, hypofractionation

## Abstract

Background: Combining external beam radiotherapy (EBRT) with high-dose-rate (HDR) brachytherapy (BT) enables biologically effective dose escalation in prostate cancer. However, comparative evaluation of such regimens using radiobiological modeling remains limited. Methods: Dose regimens based on clinical practice were analyzed using α/β values of 1.5 and 3 Gy for the prostate. Ten patients with available planning CT, pelvic MRI, and ultrasound-guided BT plans were retrospectively evaluated. Physical and biological dose distributions were recalculated for various EBRT and HDR-BT combinations. Biological effective dose (BED) values were determined for the prostate and organs at risk (OARs: anterior rectal wall, bladder base, urethra). Regimens yielding the highest ΔBED between prostate and OARs were considered most favorable. Results: All regimens met clinical dose constraints. The most favorable ΔBED profiles for bladder and rectum were observed with HDR-BT regimens (2 × 15 Gy) combined with either 23 × 2 Gy or 15 × 2.5 Gy EBRT, independent of the assumed α/β value. EBRT-only regimens achieved superior urethral sparing, while higher HDR doses led to increased urethral exposure. Conclusions: This study underscores the value of radiobiological modeling in differentiating and optimizing prostate cancer radiotherapy strategies. While the trade-offs between dose escalation and OAR sparing are clinically known, our biologically driven analysis provides a more quantitative foundation for selecting and tailoring combined EBRT/HDR-BT regimens in practice.

## Simple Summary

Radiotherapy is a key treatment for prostate cancer, and combining external beam radiotherapy (EBRT) with high-dose-rate (HDR) brachytherapy can increase treatment effectiveness. However, it remains unclear which dose combinations best balance tumor control and protection of healthy organs. In this study, we retrospectively analyzed ten prostate cancer patients who were actually treated with combined EBRT and HDR brachytherapy. Using their original treatment plans, we applied radiobiological models to compare how different combinations affected the prostate and nearby organs such as the bladder, rectum, and urethra. We found that certain combinations may offer more favorable dose distributions to the tumor while limiting exposure to critical structures. These results do not define a single best regimen but provide quantitative insights that may help guide treatment planning in clinical practice and support future research on individualized prostate cancer radiotherapy.

## 1. Introduction

Prostate cancer (PC) is the most commonly diagnosed malignancy in men worldwide [1]. In patients treated with curative intent, both radical prostatectomy and radiotherapy (RT), with or without androgen deprivation therapy (ADT), are established treatment options, offering comparable overall survival outcomes [2]. For intermediate- and high-risk PC, radiotherapy may be delivered as external beam radiotherapy (EBRT) alone or as a combination of EBRT and brachytherapy (EBRT+BT) [3,4,5]. However, the optimal approach among these modalities remains a subject of ongoing clinical investigation.

A key radiobiological parameter in prostate cancer is the α/β ratio, which is estimated to lie between 1.5 Gy and 3 Gy [6,7]. This relatively low value suggests a high sensitivity of prostate tumors to fraction size, thereby supporting the rationale for dose escalation through hypofractionated treatment regimens [8]. Delivering larger doses per fraction may enhance tumor control, particularly when using modern, highly conformal techniques that enable safe delivery of high-dose EBRT [9,10,11].

Before the emergence of stereotactic body radiotherapy (SBRT), hypofractionation had already been implemented in high-dose-rate brachytherapy (HDR-BT) as a boost in combination with EBRT [12]. This approach continues to offer a promising avenue for maximizing the therapeutic ratio by increasing tumor dose while minimizing exposure to adjacent organs at risk (OAR).

However, the optimal approach among these modalities remains a subject of ongoing clinical investigation. This reflects a broader trend in prostate cancer management toward more individualized and targeted strategies. For example, focal therapy has emerged as a middle ground between overtreatment and undertreatment, aiming to eliminate the dominant lesion while minimizing toxicity and psychological burden for patients [13].

### 1.1. High-Dose-Rate Brachytherapy (HDR-BT)

HDR-BT is an established, highly conformal hypofractionated technique in prostate cancer treatment [4,14]. Its key advantage over EBRT lies in minimizing the impact of organ motion, allowing for precise intraprostatic dose escalation and rapid dose fall-off, thereby effectively sparing surrounding organs at risk (OARs). While randomized trials comparing EBRT alone versus EBRT combined with brachytherapy remain limited, several studies have demonstrated improved biochemical control with combined treatment, attributed to higher intraprostatic doses [15,16]. Low-dose-rate (LDR) BT and HDR-BT have both shown clinical efficacy, though HDR-BT offers better control of periprostatic disease when used as a boost [17]. Clinical outcomes in intermediate- and high-risk patients treated with combined EBRT and HDR-BT are consistently favorable [18].

### 1.2. Modern EBRT Techniques: Intensity-Modulated Radiotherapy (IMRT) and Volumetric Modulated Arc Therapy (VMAT)

Dose escalation using conventional 3D-conformal RT (3D-cRT) has not consistently improved overall survival in prostate cancer, and its broader dose distribution increases the risk of OAR toxicity [19,20,21]. In contrast, modern techniques like IMRT and VMAT provide highly conformal dose delivery, reducing toxicity while maintaining oncologic effectiveness [22]. VMAT, in particular, offers advantages over IMRT, including shorter treatment times, fewer monitor units, and improved intrafractional precision [23]. These attributes make VMAT especially suitable for hypofractionation and integration with HDR-BT.

#### 1.2.1. Hypofractionated EBRT

Multiple randomized trials have shown that moderate and ultra-hypofractionated EBRT provide oncologic outcomes equivalent to conventional fractionation in localized prostate cancer [10,11,24,25,26,27]. Consequently, hypofractionation is now widely adopted [28]. However, some studies report increased rates of late gastrointestinal (GI) and genitourinary (GU) toxicities with hypofractionated regimens, underscoring the importance of careful patient selection and technique optimization [24,25,27].

#### 1.2.2. Combining Hypofractionated EBRT with HDR-BT

Recent phase I/II trials have explored the combination of hypofractionated or ultra-hypofractionated EBRT with HDR-BT, aiming to enhance tumor control while preserving tolerability [29,30,31,32]. However, the optimal regimen balancing oncologic efficacy and toxicity remains undefined. This study was conducted to systematically compare various dose concepts and identify treatment strategies that maximize intraprostatic dose while maintaining acceptable exposure to OARs.

### 1.3. Study Objectives

To better understand the optimal use of hypofractionated EBRT and HDR-BT in prostate cancer, this study retrospectively compares a wide range of clinically relevant treatment concepts with regard to their biological effectiveness and safety profiles. The analysis is divided into two parts:

Part A compares five different RT regimens—three EBRT-only and two EBRT combined with HDR-BT—under the assumption of equal biological effectiveness in the target volume, focusing on organ-at-risk exposure.

Part B investigates 15 RT regimens with varying biological doses to the target, analyzing the therapeutic ratio (ΔBED) between the prostate and adjacent OARs.

This two-part approach aims to identify treatment strategies that enable dose escalation to the prostate while minimizing toxicity risks.

## 2. Materials and Methods

### 2.1. Materials

Ten patients with histopathological confirmed intermediate- or high-risk adenocarcinoma of the prostate who received primary definitive RT at the Department of Radiotherapy and Radiooncology, University Medical Center Goettingen, Germany, were included in this study. Treatment was administered between 2019 and 2021 and consisted of a combination of EBRT (25 × 2 Gy) and HDR-BT (2 × 9 Gy). HDR brachytherapy was applied either prior to or between EBRT fractions, depending on clinical logistics. While EBRT was temporarily paused during BT delivery, this sequencing reflects real-world practice and did not influence the dosimetric or biological modeling outcomes, which were based on completed treatment plans. To evaluate changes in prostate volume due to HDR-BT, an EBRT treatment planning computed tomography (P-CT) scan was repeated three to four days after the second HDR-BT to allow adjustment of the clinical target volume (CTV).

#### 2.1.1. Treatment Planning

Administered doses and dose constraints in studies on conventional and hypofractionated EBRT ± HDR-BT for prostate cancer vary considerably. To compare dose concepts involving hypofractionated VMAT-EBRT ± HDR-BT, various combinations were calculated. Based on Zelefsky et al., a target dose of 43 × 2 Gy (86 Gy) was selected [33]. Equivalent doses in 2 Gy fractions (EQD2) and BED were calculated using α/β ratios of 1.5 and 3 for prostate tissue (Table 1, Supplemental Table S1). In the second step, these hypothetical concepts were translated into clinically applicable dose regimens (Table 2).

#### 2.1.2. EBRT VMAT Technique

Pelvic EBRT planning CT scans (Brilliance CT Big Bore Oncology, Philips, Amsterdam, Netherlands) were acquired in 3 mm slices and transferred to the contouring software (Varian Eclipse V15.6, Varian Medical Systems, Palo Alto, CA, USA). A pelvic magnetic resonance imaging (MRI) was performed and registered according to departmental standard operating procedures. The planning target volume (PTV) was defined as the CTV plus a 5 mm isotropic margin. For better comparability with HDR-BT, and in contrast to routine clinical practice, the delineation OARs was modified as follows: the anterior rectal wall was contoured from 6 mm cranial to 6 mm caudal of the PTV; the bladder was contoured as the 2 cm segment adjacent to the PTV. The urethra was identified on the planning MRI and expanded by a 4 mm radius. VMAT plans were created using 6 MV photon beams delivered via full arcs on a Varian Clinac 2300 linear accelerator equipped with a 120-leaf Millennium multileaf collimator (MLC). To minimize the MLC tongue-and-groove effect, 0° gantry angles were avoided. The isocenter was placed at the center of the PTV. Planning and optimization were performed using Eclipse V15.6 with the AcurosXB algorithm.

##### Dose Constraints—EBRT VMAT

For VMAT-EBRT, dose constraints for the rectum and bladder were based on the phase II feasibility study NCT01982786 [5]. Constraints for unspecified fractionation schemes were scaled based on EQD2-adjusted daily doses (Supplemental Tables S2–S6). Urethral dose limits followed published clinical trials (DELINEATE; [34]). For ultra-hypofractionated regimens (Table 2), constraints were based on recent comparative studies [35]. Dose coverage followed the International Commission on Radiation Units and Measurements (ICRU) reports 50, 62, and 83 for 2 Gy per fraction. Normo- as well as hypofractionated regimens were prescribed to the 95% isodose.

#### 2.1.3. HDR-BT Technique

HDR-BT planning was performed based on ultrasound-guided catheter placement, using the Oncentra Prostate Treatment Planning System (Version 4.2.21, Varian Medical Systems). The CTV was defined as the prostate without seminal vesicles, PTV was equivalent to the CTV. OARs included the anterior rectal wall, bladder neck, and urethra. The anterior rectal wall was contoured from 6 mm cranial to 6 mm caudal of the CTV. The urethra was contoured from the Foley catheter balloon in the bladder to the inferior edge of the CTV, with an additional 4 mm radial margin for consistency with EBRT planning. The bladder was contoured as the 2 cm segment adjacent to the CTV, receiving the highest brachytherapy dose. The source step size was 5 mm. Dose distribution was optimized manually. HDR-BT was delivered using a remote afterloader with an iridium-192 source (Nucletron microSelectron HDR^®^, Elekta, Stockholm, Sweden until June 2020; Flexitron HDR^®^, Elekta, from July 2020 onward).

##### Dose Constraints—HDR-BT

HDR-BT dose constraints were: ≥90% of the prescribed dose (PD) to ≥100% of the CTV (D90% ≥ 100%); V150% < 35%; V200% < 15%. The volume of the anterior rectal wall and bladder neck receiving 75% of the dose was limited to <1 cm^3^ (V75% < 1 cm^3^). D0.1ccm for the bladder neck was limited to <115% of PD; for the urethra, D0.1ccm < 120%, and V120% < 20%. Further constraints are detailed in Supplemental Table S7.

#### 2.1.4. Evaluation of VMAT and HDR-BT Planning Combinations

Retrospective comparative planning was performed. Dose–volume histograms (DVHs) were generated for the target volumes (EBRT: PTV; HDR-BT: CTV) and the anterior rectal wall, bladder neck, and urethra. In the first step (Study Part A), various dose prescriptions for conventional and hypofractionated VMAT with or without HDR-BT boost were modeled. EQD2 values were calculated for α/β = 1.5 and 3 for prostate tissue (Table 1, Supplemental Table S1). For OARs, α/β ratios were: rectum = 3, bladder = 2, and urethra = 1 [36,37,38]. Treatment plans to deliver a PTV EQD2 equivalent to 86 Gy normofractionated VMAT were created, and doses to OARs were evaluated. In a second step (Study Part B), HDR-BT + VMAT and VMAT-only dose–volume concepts were compared for dose escalation potential while sparing OARs (Table 2).

DVH parameters for PTV converted to EQD2 were: mean EQD2, D90%, and D95% (Table S6). For OARs, the following EQD2-converted metrics were analyzed: Rectum and bladder: D2cc, D1cc, D0.1cc; Urethra: D30%, D10%, D1cc, D0.1cc, and Dmin.

### 2.2. Study Part A

In this part of the study, five different radiotherapy regimens were compared—three involving exclusively external beam radiotherapy (EBRT) and two combining EBRT with brachytherapy. The dose prescriptions were selected to ensure equivalence in biological effectiveness within the planning target volume (PTV), corresponding to an EQD2 of 86 Gy in all cases. For each of the five regimens, BED values were calculated for three organs at risk (OARs): the urinary bladder (assuming α/β = 2.0), rectum (α/β = 3.0), and urethra (α/β = 1.0). For the bladder and rectum, dose exposure was evaluated using the volumetric metrics D_2_ccm, D_1_ccm, and D_0_._1_ccm, while for the urethra, D_1_ccm, D_0_._1_ccm, and Dmin (the minimum dose) were assessed.

Each of the five regimens was analyzed pairwise for ten patients to determine the most favorable BED exposure to the OARs. Statistical comparisons were performed using the non-parametric, paired Wilcoxon signed-rank test. A *p*-value of <0.05 was considered nominally statistically significant; no correction for multiple comparisons was applied, given the exploratory nature of the analysis. Additionally, the median BED values across the ten patients were summarized using box plots, displaying interquartile ranges, minimum, maximum, and—where applicable—outliers and extreme values. To account for the risk of false-positive findings due to multiple tests, we additionally performed a false discovery rate (FDR) correction for all pairwise comparisons of dose–volume parameters in Study Part A. The FDR-adjusted *p*-values are provided alongside the raw *p*-values in the Supplemental Excel File S1 “*p* values_organs at risk_study part A”.

### 2.3. Study Part B

This part included 15 radiotherapy regimens: six EBRT-only and nine combining EBRT with HDR-BT. In contrast to Part A, EQD2 values to the clinical target volume (CTV, i.e., the prostate) varied between regimens. Therefore, Part B focused on analyzing BED differentials between the prostate and the specified OARs.

To account for radiobiological uncertainty in prostate cancer, BED calculations for the CTV were performed using two α/β ratios: 1.5 Gy, reflecting the commonly assumed low fractionation sensitivity, and 3 Gy, representing a more conservative alternative based on interpatient variability. Initial calculations were based on α/β = 1.5 Gy, followed by comparative analysis using α/β = 3 Gy to evaluate the robustness of results. For OARs, the same α/β values as in Part A were applied.

As in Part A, pairwise comparisons between regimens were conducted using the paired Wilcoxon signed-rank test, applying the same threshold for nominal statistical significance (*p* < 0.05) and again without correction for multiple testing. Differences in BED between the target volume and each OAR (ΔBED) were also analyzed and visualized using box plots, including median, interquartile range, minimum, maximum, and, where applicable, outliers and extreme values. A greater ΔBED indicates a more favorable therapeutic ratio, i.e., a higher relative dose to the target volume compared to the OARs.

## 3. Results

First, we analyzed dose parameters at OARs for fixed given doses in the PTV (α/β ratio for the prostate of 1.5: Table 1, α/β ratio for the prostate of 3: Supplemental Table S1).

**Figure 1:**
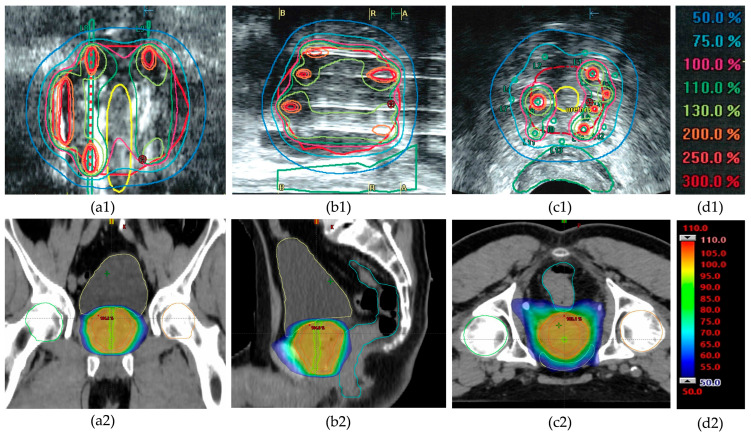
Example of dose distribution in high-dose-rate (HDR) brachytherapy and external beam radiotherapy (EBRT) for prostate cancer treatment of patient #6. Top row (**a1**–**c1**): Ultrasound-based images showing dose distribution in HDR brachytherapy with color-coded isodose lines (see color legend (**d1**)). Coronal (**a1**), sagittal (**b1**), and transverse (**c1**) views illustrate highly heterogeneous intraprostatic dose distributions with focal high-dose regions (>200% of the prescribed dose). Note the sparing of the urethra (yellow contour in (**a1**,**c1**)) and the rectum (green contour in (**b1**,**c1**)). Bottom row (**a2**–**c2**): CT-based dose distribution in EBRT using the VMAT technique in corresponding planes, showing a more homogeneous dose coverage around the prescribed target dose (color legend (**d2**)). Note that organs at risk receive a higher percentage of the prescribed dose (urethra: green; rectum: blue; bladder: yellow).

**Figure 2:**
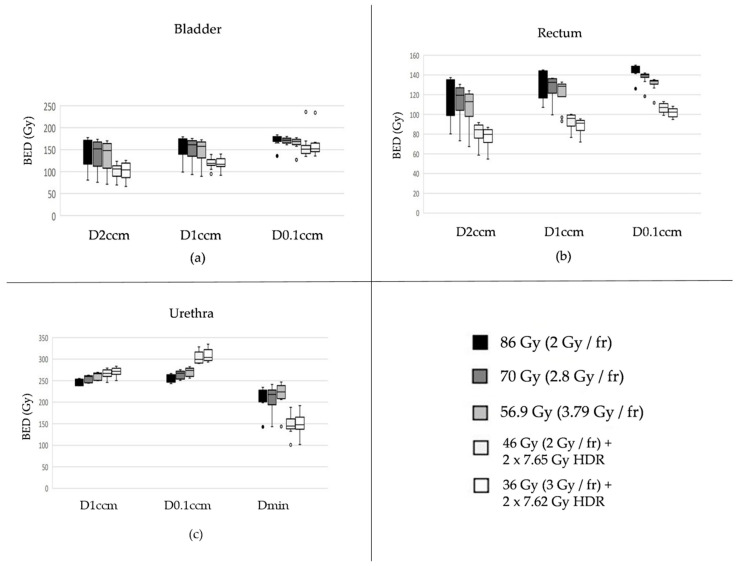
Biologically effective doses (BEDs) for the three most exposed subvolumes of the bladder (**a**), rectum (**b**), and urethra (**c**) are shown across different fractionation schemes, all normalized to an equivalent BED in the prostate target assuming an α/β ratio of 1.5 Gy. Organ-specific α/β values were assumed as follows: 2.0 Gy for the bladder, 3.0 Gy for the rectum, and 1.0 Gy for the urethra. Description of the box plots: The thick horizontal line inside the rectangular box indicates the median of the data distribution. The lower edge of the gray-shaded box represents the first quartile (Q1), and the upper edge represents the third quartile (Q3). The vertical length of the box (i.e., Q3–Q1) is known as the interquartile range (IQR). The whiskers on either side extend to values up to 1.5 times the IQR from Q1 or Q3. Data points located more than 1.5 but not more than 3.0 times the IQR from Q1 or Q3 are considered outliers (depicted as circles), while those farther than 3.0 times the IQR are classified as extreme values (typically shown as asterisks—none appear here).

The two radiation regimens comprised of combinations of percutaneous and interstitial radiation therapies resulted in statistically significantly lower bladder radiation exposure compared to the three modalities consisting of solely EBRT (*p* values each <0.01 for six pairwise comparisons referring to the most exposed 2 and 1 ccm, respectively, Figure 1(a1,a2), left and middle). The two procedures with BT exhibited a statistical trend in favor of the condition with 36 à 3 Gy combined with HDR 2 × 7.62 Gy when compared with 46 à 2 Gy with HDR 2 × 7.65 Gy (*p* = 0.07 for both D2ccm and D1ccm). Regarding the most exposed 0.1 ccm, distinctions were less pronounced, albeit still featuring the two combined modalities favorable over the three percutaneous applications (Figure 2a, right).

Similar to the bladder, comparable patterns were observed for the rectum, with both combination therapies again demonstrating a more favorable profile in terms of reduced radiation exposure to this organ at risk (Figure 2b).

Unlike for bladder and rectum, the two modalities comprising EBRT- and BT came along with the highest exposures for D1ccm and particularly D0.1ccm for the Urethra (Figure 2c). For the latter, combined therapies exhibited all with *p* < 0.01 in pairwise comparisons with the three percutaneous treatments only. On the contrary, Dmin values elicited favorable for the two combination regimens, both with pairwise *p* values < 0.01 compared to each of the three EBRT only.

In Study Part B, we applied radiation regimens that differed in terms of the BEDs at the PTV (Table 2), calculated separately using α/β values of 1.5 and 3.0. These were then used to compare the corresponding BEDs delivered to the OAR, based on α/β values of 2 for the urinary bladder, 3 for the rectum, and 1 for the urethra [36,38]. The various regimens all fulfilled the predefined dose constraints both at the PTV and the organs at risk. Thus, the most effective strategies were considered to have the largest BED differences between the PTV and the OAR.

**Figure 3:**
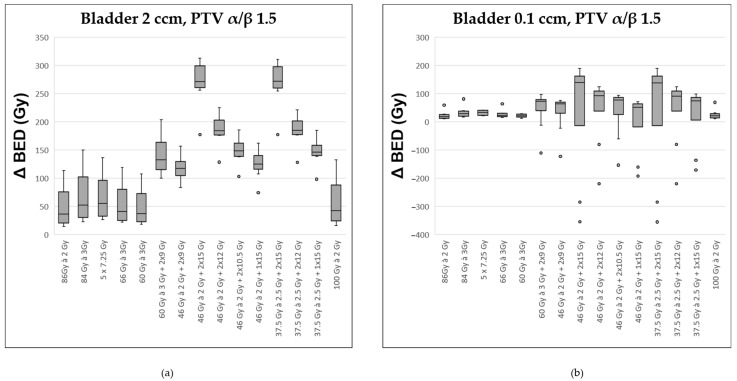
Comparison of various fractionation schemes regarding the difference in biologically effective dose (ΔBED) between the target prostate volume (assuming an α/β ratio of 1.5) and the most exposed portion of the bladder (α/β ratio of 2.0): (**a**) referring to the highest exposed 2 ccm, (**b**) to the highest exposed 0.1 ccm. A detailed explanation of the boxplots is provided in the legend of Figure 2.

Assuming an α/β-ratio of 1.5 for the prostate, the most pronounced differences between the BED at the PTV and the BED at the most exposed 2 cc of the bladder were observed. These occurred with the two regimens containing the highest proportion of HDR-BT: 46 × 2 Gy + HDR 2 × 15 Gy and 37.5 × 2.5 Gy + HDR 2 × 15 Gy (Figure 3a). Those schedules without any BT exhibited the lowest gradient between the dose in the PTV and that in the hottest 2 ccm of the bladder. Comparable results were observed when considering the most exposed 1 cc of the bladder. 

Regarding the most exposed 0.1 ccm of the bladder, distinctions between the various radiation regimens were less pronounced (Figure 3b). The two regimens incorporating HDR 2 × 15 Gy continued to show the most favorable median difference between the dose at the PTV and that at the most exposed 0.1 cc of the bladder. However, across the series of ten modeled patients, there was considerable variability, preventing a clear advantage for any single treatment approach.

Assuming an α/β ratio of 3.0 for the prostate revealed similar BED relationships between the PTV and the bladder for the considered treatment modalities. The most beneficial BED differences again were noticed for the two regimens with an HDR component of 2 × 15 Gy. For instance, the situation is illustrated for the most exposed 2 ccm volume of the bladder (Supplemental Figure S1).

With regard to the rectum, findings were even more consistent compared to the bladder. In addition to the 1 and 2 ccm of the most exposed areas, in the rectum also the volume of 0.1 ccm clearly showed the superiority of the two treatments containing two HDR courses of 15 Gy each (Supplemental Figure S2). Almost identical relationships were observed based on an α/β-ratio of 3.0 for the prostate PTV. Thus, highly uniform results were obtained for the rectum irrespective of the considered dose volumes or independent from the α/β assumed for the prostate target volume.

**Figure 4:**
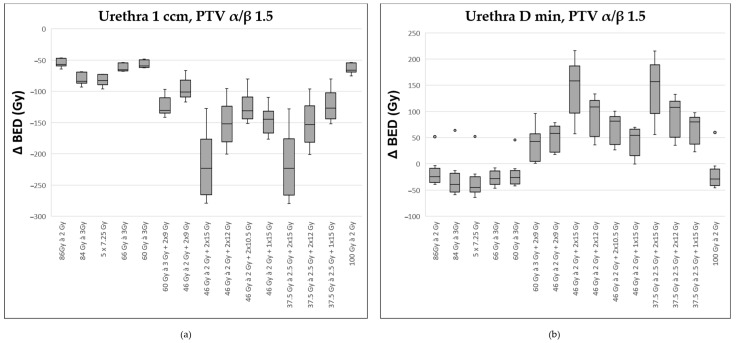
Comparison of various fractionation schemes regarding the difference in biologically effective dose (ΔBED) between the target prostate volume (assuming an α/β ratio of 1.5) and the most exposed portion of the urethra (α/β ratio of 1.0): (**a**) referring to the highest exposed 1 ccm, (**b**) to the minimally exposed volume (Dmin) 0.1 ccm. A detailed explanation of the boxplots is provided in the legend of Figure 2.

With respect to the urethra, the situation is presented differently. The two regimens with 2 × 15 Gy HDR were associated with the most unfavorable Δ BED for the maximal exposed areas (D 1ccm and 0.1 ccm; exemplarily illustrated for D 1 ccm, Figure 4a). However, when considering the minimal dose to which the urethra was exposed, the modalities with 2 × 15 Gy HDR turned out favorable compared to the other regimens (see Figure 4b).

Assuming an α/β of 3.0 for the prostate elicited similar results for the Δ BED for the urethral volumes of 1 and 0.1 ccm. In contrast to the α/β of 1.5, the two simulated treatments with 2 × 15 Gy HDR here did not show more favorable in terms of D min.

## 4. Discussion

The combination of modern radiotherapy techniques such as EBRT and HDR-BT offers the potential to escalate the therapeutic dose within the prostate while maintaining acceptable toxicity levels for surrounding organs. Due to the low α/β ratio of prostate cancer (1.5–3 Gy), hypofractionated approaches may provide a therapeutic advantage [17,39]. Our study evaluated different combinations of normo, hypo-, and ultrahypofractionated EBRT with HDR-BT in terms of dose escalation and OAR sparing. Unlike prior studies such as Chatzikonstantinou et al., which created virtual HDR-BT plans based on TRUS images, our analysis relied on actual delivered HDR-BT plans based on ultrasound-guided catheter placement, reflecting real clinical practice rather than idealized implant geometry [35]. Image guidance in our study combined TRUS for HDR-BT planning with CT and MRI for EBRT planning. This multimodal approach enabled a robust comparison of prostate volumes. No significant differences were found between volumes measured via TRUS and those derived from MRI/CT, supporting their comparability. Several studies have confirmed the strong agreement between prostate volumes measured by TRUS, MRI, and pathological specimens [40,41,42].

Our analysis demonstrated that treatment regimens including HDR-BT resulted in significantly lower doses to the bladder and rectum compared to purely external beam approaches while achieving higher intraprostatic dose coverage. This held true across both α/β assumptions. However, urethral dose was consistently higher with HDR-BT combinations, highlighting it as a critical dose-limiting factor [43,44,45]. The most favorable ΔBED between the PTV and OARs was observed in regimens with the highest HDR-BT contribution (e.g., 46 Gy in 2.0 Gy fractions or 37.5 Gy in 2.5 Gy fractions, each combined with 2 × 15 Gy HDR-BT). These protocols offer the most pronounced intraprostatic dose escalation, albeit at the cost of urethral toxicity. Studies such as RTOG 0321 and Ghadjar et al. have shown that urethral doses >120% of the HDR-BT prescriptions are associated with increased acute and late GU toxicity [43,46,47]. In terms of rectal and bladder toxicity, dose escalation has long been known to increase GI and GU side effects. RTOG 0126 and GETUG 06 both reported increased ≥ grade 2 toxicities with higher total doses [19,48]. Although the CHHiP trial found no significant difference in toxicity between fractionation schemes, it proposed constraints (e.g., V60 Gy < 0.01%) that are difficult to achieve in daily practice due to the close proximity of the prostate to the rectum [26]. Perirectal hydrogel spacers have proven effective in reducing rectal dose and associated toxicity, as confirmed in conventional and stereotactic regimens [49,50]. For the bladder, increasing evidence suggests that it behaves as a serial organ, sensitive to high focal doses. Recent studies have shown correlations between specific bladder dose exposures and the development of acute and late GU toxicity [51,52].

When placed in the context of current guideline recommendations, both ASTRO and ESTRO endorse HDR brachytherapy (HDR-BT) boost as a standard treatment option for intermediate- and high-risk prostate cancer. The ESTRO/EAU/EORTC consensus guideline (2020) recommends HDR-BT boosts with total doses of 5–15 Gy per fraction, typically administered in 1–3 fractions, in combination with EBRT regimens of 45–50.4 Gy in 1.8–2 Gy fractions [18,53]. The ASTRO guideline similarly supports EBRT combined with a brachytherapy boost, citing evidence of improved biochemical control.

Combining hypofractionated EBRT with HDR-BT is particularly promising, as it enables delivery of high total doses over a shorter treatment period—potentially improving patient convenience and reducing healthcare costs. Our findings support this approach and are consistent with emerging clinical evidence. For instance, the SPARE trial used 1 × 15 Gy HDR-BT combined with 5 × 5 Gy pelvic EBRT in high-risk patients, reporting a 5-year biochemical control rate of 81.1% with acceptable toxicity [32]. Similarly, Gomes-Iturriaga et al. tested a comparable approach with favorable safety outcomes [54].

In comparison, our modeled regimens represent biologically plausible dose combinations that align with these clinical trends toward treatment intensification and reduced overall duration. Although our modeling does not allow direct prediction of tumor control, the estimated doses to the prostate and surrounding tissues fall within—or even exceed—the ranges used in the cited trials. This suggests that similar clinical outcomes might be achievable, though this remains to be confirmed in prospective studies.

It is also worth noting that the bladder and rectum exhibit distinct radiobiological characteristics, including relatively low α/β ratios as well as pronounced volume and dose-rate effects. These properties affect their sensitivity to hypofractionation and should be considered when interpreting dose–volume parameters across different fractionation regimens. These early clinical results, in combination with our model-based analysis, highlight the need for further trials to systematically evaluate the efficacy, safety, and practicality of such combined treatment approaches.

Our proposed combinations are consistent with these recommendations but reflect a growing trend toward moderate hypofractionation in the EBRT component. The use of 15 × 2.5 Gy (total 37.5 Gy) represents a biologically equivalent dose close to the conventional 45–50 Gy range when combined with HDR-BT while reducing overall treatment time. Although such regimens are not yet universally adopted, they align with the flexibility allowed in current guidelines, particularly in centers with strong brachytherapy expertise. Thus, our findings provide quantitative support for hypofractionated HDR-BT-based schedules within the framework of existing standards. A key limitation of our study is the small sample size (*n* = 10), which limits statistical power and generalizability. Consequently, the analysis is descriptive in nature and not intended to establish statistical significance in the conventional sense. While our analyses were exploratory in nature, we addressed the concern of type I errors by applying an FDR correction in a sensitivity analysis. The results remained largely unchanged, supporting the robustness of our findings; however, the possibility of false-positive results cannot be entirely excluded. The consistent imaging, contouring, and planning methodology across all cases provides a solid foundation for biologically meaningful comparisons of dose regimens. These controlled conditions allow for robust insight into relative dose distributions and trade-offs between tumor coverage and OAR sparing. Future studies with larger patient cohorts should aim to validate and expand upon these findings using formal statistical methods, particularly to account for interindividual variability.

In addition, the inclusion of hypothetical EBRT-only regimens that are not part of the current clinical routine warrants clarification. These high-dose strategies (e.g., 100 Gy at 2 Gy/fraction or 84 Gy at 3 Gy/fraction) were deliberately incorporated as radiobiological reference points to explore the theoretical upper limits of EBRT-based dose escalation. Their inclusion enabled a more comprehensive and quantitative comparison with HDR-BT boost combinations and helped to illustrate the favorable balance such combinations can offer—achieving comparable or higher prostate BED while maintaining superior sparing of organs at risk. Although clinical outcome data from our cohort are not available due to the dosimetric nature of the study, the findings may serve as a foundation for hypothesis-driven prospective trials. Such studies could investigate whether the dosimetric advantages observed—particularly regarding organ-at-risk sparing with HDR-BT boosts—translate into improved toxicity profiles and biochemical control.

Beyond these specific comparisons, our study highlights the broader potential of radiobiological modeling as a decision-support tool in modern radiotherapy planning. By incorporating different α/β values, we demonstrate how biological assumptions influence regimen assessment and may ultimately affect clinical decisions. Looking ahead, radiobiological modeling could play a key role in the development of personalized treatment strategies, guiding dose-escalation protocols, supporting patient selection based on individual radiosensitivity, and improving the balance between efficacy and toxicity. Its integration into clinical workflows could thus contribute meaningfully to the refinement and personalization of prostate cancer radiotherapy.

In this context, genomic classifiers such as Decipher^®^ and Prolaris^®^ offer a promising complement to dosimetric optimization. These tools enable a biologically informed risk stratification that could support treatment intensity decisions—e.g., identifying patients who may benefit from dose escalation via HDR-BT boosts versus those eligible for de-escalated EBRT approaches. Although their adoption in routine clinical practice remains limited, recent data underscore a growing interest in their application [55]. Integrating such molecular insights into radiotherapy planning may enhance personalization beyond anatomical and radiobiological parameters alone, further refining the therapeutic ratio.

## 5. Conclusions

Our study provides a detailed radiobiological and dosimetric comparison of various EBRT and HDR-BT combination regimens for prostate cancer. The results highlight the potential of HDR-BT boosts to achieve significant intraprostatic dose escalation while reducing rectal and bladder exposure compared to EBRT-only approaches. However, urethral dose remains a critical limiting factor. Among the investigated regimens, those with a higher HDR-BT contribution offered the most favorable therapeutic ratio in terms of tumor coverage versus organ-at-risk sparing.

Although limited by the small cohort size, the controlled methodology and consistent planning approach allow for meaningful comparative insights. Our findings support the further exploration of combined hypofractionated EBRT and HDR-BT regimens and underscore the value of radiobiological modeling as a tool to guide individualized treatment strategies. Future studies should validate these results in larger cohorts and investigate the integration of genomic risk profiling to optimize treatment intensity and personalization.

## Figures and Tables

**Table 1:** Different dose prescriptions for conventional and hypofractionated VMAT plans with or without an HDR BT boost and corresponding biologically equivalent dose to organs at risk; α/β ratio for prostate of 1.5, converted to an EQD2 of 86Gy. Organ-specific α/β values were assumed as follows: 2.0 Gy for the bladder, 3.0 Gy for the rectum, and 1.0 Gy for the urethra.

		Prostate α/β 1.5 Gy						Biologically Equivalent Dose: Median (Q1-Q3)								
		EQD2 Values			BED Values			Dmin	D0.1ccm			D1ccm			D2ccm	
Radiation Technique	Dose (Gy)	VMAT	HDR	Total	VMAT	HDR	Total	Urethra	Rectum	Bladder	Urethra	Rectum	Bladder	Urethra	Rectum	Bladder
VMAT	86 (43 × 2)	86		86	200.67		200.67	215.1 (204.2–225.6)	148.3 (143.6–148.6)	175.6 (170.2–179.0)	253.5 (246.6–262.5)	140.4 (122.7–143.5)	165.3 (144.6–172.7)	251.8 (239.1–253.4)	125.1 (103.7–135.0)	156.0 (126.3–167.9)
VMAT hfx	70 Gy (25 × 2.8)	86		86	200.67		200.67	217.9 (203.6–225.1)	140.2 (139.0–140.8)	171.6 (166.4–175.3)	267.0 (254.7–271.1)	132.5 (128.2–135.6)	161.2 (140.0–168.5)	259.8 (245.9–261.2)	119.4 (109.4–126.8)	151.7 (121.3–163.7)
VMAT hfx	56.9 (15 × 3.8)	86		86	200.71		200.71	224.0 (211.7–236.3)	133.8 (132.3–134.1)	168.9 (163.1–172.6)	273.6 (260.6–277.9)	128.5 (125.5–130.2)	157.7 (136.3–165.3)	265.9 (251.5–267.6)	112.9 (103.1–120.4)	148.0 (117.2–120.6)
VMAT nfx + HDR	46 Gy (23 × 2) + 2×7.7	46	40	86	107.33	93.33	200.66	144.5 (140.7–151.1)	107.0 (102.4–110.1)	151.1 (144.1–155.8)	299.5 (292.5–314.8)	95.9 (89.6–99.1)	118.7 (117.1–124.6)	266.9 (261.7–273.6)	84.5 (78.0–88.9)	106.1 (94.8–110.9)
VMAT hfx + HDR	36 (12 × 3) + 2 × 7.6	46.29	39.7	86	108	92.66	200.66	147.8 (139.5–155.6)	102.3 (97.7–105.0)	151.8 (147.1–161.6)	303.6 (298.4–320.2)	91.1 (85.1–93.5)	116.7 (114.9–127.4)	271.6 (266.3–277.3)	79.7 (73.3–84.1)	104.1 (91.7–116.0)

Abbreviations: EQD2 = equivalent dose to 2 Gy/fraction; BED = biologically equivalent dose; VMAT: volumetric modulated arc therapy; HDR: high dose rate brachytherapy, Gy: Gray; fr = fraction.

**Table 2:** Dose concepts for conventional and hypofractionated VMAT plans with or without an HDR BT boost; α/β ratio for prostate of 1.5 and 3.

	EQD2 Values (α/β = 1.5 Gy) in Gy			BED Values (α/β = 1.5 Gy) in Gy			EQD2 Values (α/β = 3 Gy) in Gy			BED Values (α/β = 3 Gy) in Gy		
Radiation Technique	VMAT	HDR	Total	VMAT	HDR	Total	VMAT	HDR	Total	VMAT	HDR	Total
Normofractionated VMAT												
86 Gy / 43 fr (2 Gy/fr)	86	-	86	206.7	-	206.7	86	-	86	143.33	-	143.33
100 Gy / 50 fr (2 Gy/fr)	100	-	100	233.3	-	233.3	100	-	100	166.76	-	166.76
Moderately hypofractionated VMAT												
60 Gy / 20 fr (3 Gy/fr)	77.14	-	77.14	180	-	180	72	-	72	120	-	120
66 Gy / 23 fr (3 Gy/fr)	84.86	-	84.86	198	-	198	79.2	-	79.2	132	-	132
84 Gy / 28 fr (3 Gy/fr)	108	-	108	252	-	252	100.8	-	100.8	168	-	168
Ultra-hypofractionated VMAT												
36.25 Gy / 5 fr (7.25 Gy/fr)	90.63	-	90.63	211.46	-	211.46	74.31	-	74.13	123.85	-	123.85
Normofractionated VMAT + HDR												
46 Gy / 23 fr (2 Gy/fr) VMAT + 2 × 9 Gy HDR	46	54	100	107.33	126	233.33	46	43.2	89.2	76.67	72	148.67
46 Gy / 23 fr (2 Gy/fr) + 2 × 10.5 Gy HDR	46	72	118	107.33	168	275.33	46	56.7	102.7	76.67	94.5	171.17
46 Gy / 23 fr (2 Gy/fr) + 2 × 12 Gy HDR	46	92.57	138.57	107.33	216	323.33	46	72	118	76.67	120	196.67
46 Gy / 23 fr (2 Gy/fr) + 2 × 15 Gy HDR	46	141.4	187.4	107.33	330	437.33	46	108	154	76.67	180	256.67
46 Gy / 23 fr (2 Gy/fr) + 1 × 15 Gy HDR	46	70.71	116.71	107.33	165	272.33	46	54	100	76.67	90	166.67
Moderately hypofractionated VMAT + HDR												
37.5 Gy / 15 fr (2.5 Gy/fr) + HDR 2 × 15 Gy	42.9	141.4	184.3	100	330	430	41.3	108	149.3	68.75	180	248.75
37.5 Gy / 15 fr (2.5 Gy/fr) + HDR 1 × 15 Gy	42.9	70.7	113.6	100	115	215	41.3	54	95.3	68.75	90	158.75
37.5 Gy / 15 fr (2.5 Gy/fr) + HDR 2 × 12 Gy	42.9	92.57	135.47	100	216	316	41.3	72	113.3	68.75	120	188.75
60 Gy / 20 fr (3 Gy/fr) + HDR 2 × 9 Gy	77.1	54	131.1	180	126	306	72	43.2	115.2	168	72	240

Abbreviations: EQD2 = equivalent dose to 2 Gy/fraction; BED = biologically equivalent dose; VMAT: volumetric modulated arc therapy; HDR: high dose rate brachytherapy, Gy: Gray; fr = fraction.

## Data Availability

The datasets generated and/or analyzed in the current study are available from the corresponding author upon reasonable request.

## Supplementary Materials

The following supporting information can be downloaded at: https://www.mdpi.com/article/10.3390/curroncol32060360/s1, Figure S1: Comparison of various fractionation schemes for the difference in biologically effective dose (Δ BED) between that for the target prostate volume (α/β of 3.0 assumed) and that for the highest exposed 2 ccm of the bladder (α/β of 2.0).; Figure S2: Comparison of various fractionation schemes for the difference in biologically effective dose (Δ BED) between that for the target prostate volume (α/β of 1.5 assumed) and that for the highest exposed 0.1 ccm of the rectum (α/β of 3.0); Table S1: Different dose prescriptions for conventional and hypofractionated VMAT plans with or without an HDR BT boost and corresponding biologically equivalent dose to organs at risc; α/β ratio for prostate of 3, converted to an EQD2 of 86Gy. Organ-specific α/β values were assumed as follows: 2.0 Gy for the bladder, 3.0 Gy for the rectum, and 1.0 Gy for the urethra.; Table S2: Target and organs at risk dose constraints for Table 1 + Table 2; Table S3: Target and organs at risk dose constraints for normofractionated VMAT > 80 Gy; Table S4: Target and organs at risk dose constraints for moderately hypofractionated; Table S5: Target and organs at risk dose constraints for normofractionated VMAT 46Gy; Table S6: Target and organs at risk dose constraints for ultra- hypofractionated VMAT; Table S7: Target and organs at risk dose constraints for HDR-BT; Supplemental Excel File S1: *p* values_organs at risk_study part A.

## Abbreviations

The following abbreviations are used in this manuscript:

ADT	Androgen Deprivation Therapy
BED	Biological Equivalent Dose
BT	Brachytherapy
CTV	Clinical Target Volume
DVH	Dose–Volume Histogram
EBRT	External Beam Radiotherapy
EQD2	Equivalent Dose in 2 Gy fractions
fr	Fraction
GI	Gastrointestinal
GU	Genitouriary
Gy	Gray
HDR	High-Dose-Rate
ICRU	International Commission on Radiation Units and Measurements
IMRT	Intensity-Modulated Radiotherapy
LDR	Low-Dose-Rate
MLC	Multileaf Collimator
MRI	Magnetic Resonance Imaging
OARs	Organs At Risk
PC	Prostate Cancer
P-CT	Planning Computed Tomography
PD	Prescribed Dose
PTV	Planning Target Volume
SBRT	Stereotactic Body Radiotherapy
VMAT	Volumetric Modulated Arc Therapy
3D-cRT	3D-Conformal RT
