# Supplementary material for: Dosimetric Comparison of VMAT Alone and VMAT with HDR Brachytherapy Boost Using Clinical and Biological Dose Models in Localized Prostate Cancer
Source: Curr Oncol. 2025 Jun 19;32(6):360. doi: 10.3390/curroncol32060360 (PMC12191729; doi:10.3390/curroncol32060360)
Supplement: Supplementary file 1 [file curroncol-32-00360-s001.zip › curroncol-3636554-Supplemental Tables and Figures.pdf]

Supplemental Table S1. Different dose prescriptions for conventional and hypofractionated VMAT plans with or without an HDR BT boost and corresponding biologically equivalent dose to organs at risc; α/β ratio for prostate of 3, converted to an EQD2 of 86Gy. Organ-specific α/β values were assumed as follows: 2.0 Gy for the bladder, 3.0 Gy for the rectum, and 1.0 Gy for the urethra.

		prostate α/β 3 Gy						Biologically equivalent dose: median (Q1-Q3)								
		EQD2 values			BED values			Dmin	D0.1ccm			D1ccm			D2ccm	
Radiation Technique	Dose (Gy)	VMAT	HDR	Total	VMAT	HDR	Total	Urethra	Rectum	Bladder	Urethra	Rectum	Bladder	Urethra	Rectum	Bladder
VMAT	86 (43*2)	86		86	143.3	-	143.3	215.1 (204.2-225.6)	148.3 (143.6-148.6)	175.6 (170.2-179.0)	253.5 (236.6-262.5)	140.4 (122.7-143.5)	165.3 (144.6-172.7)	251.8 (239.1-253.4)	125.1 (103.7-135.0)	156.0 (126.3-167.9)
VMAT hfx	70Gy (25*2.8)	86		86	142.5	-	142.5	234.9 (222.7-247.5)	148.7 (147.0-149.0)	182.7 (176.7-186.5)	285.8 (272.5-290.1)	140.4 (135.8-143.5)	171.4 (148.7-179.3)	277.7 (262.9-279.2)	126.4 (115.7-134.4)	161.0 (128.9-174.3)
VMAT hfx	56,9 (15*3.8)	84		84	140.5	-	140.5	248.5 (235.2-262.0)	148.9 (147.4-149.2)	187.6 (181.0-191.7)	304.1 (288.7-308.8)	140.3 (135.5-143.7)	175.5 (151.4-183.9)	294.8 (279.1-296.5)	125.9 (114.8-133.9)	164.5 (130.6-178.5)
VMAT nfx + HDR	46Gy (23*2) + 2*7.7	46	40	86	76.7	66.5	143.2	148.6 (139.3-162.7)	111.3 (106.8-116.5)	168.8 (162.6-186.0)	337.8 (327.5-358.3)	98.1 (93.1-104.3)	129.8 (124.6-137.5)	296.2 (284.7-304.1)	88.2 (81.7-91.8)	113.7 (101.4-124.9)

Supplemental Table S2. Target and organs at risk dose constraints for Table 1 + 2

Structure	Optimization parameter	Literature
Planning target volume (PTV)	PTV minimum ≥ 95%<br>PTV maximum ≤ 107%	
Anterior rectal wall	72.8Gy a´ 2.91Gy//63.7Gy a´3.749Gy<br>D15 ≤ 69.6Gy (15% volume ≤ 69.6Gy)<br>D25 ≤ 60Gy (25% volume ≤ 60Gy)<br>D35 ≤ 55.2Gy (35% volume ≤ 55.2Gy)<br>D50 ≤ 45.6Gy (50% volume ≤ 45.6Gy)<br><br>35.3Gy a´3.527Gy<br>D15 ≤ 37.1Gy (15% volume ≤ 37.1Gy)<br>D25 ≤ 32Gy (25% volume ≤ 32Gy)<br>D35 ≤ 28.8Gy (35% volume ≤ 28.8Gy)<br>D50 ≤ 34.3Gy (50% volume ≤ 34.3Gy)<br><br>70Gy a´2.8Gy<br>D15 ≤ 65.5Gy (15% volume ≤ 65.5Gy)<br>D25 ≤ 56.5Gy (25% volume ≤ 56.5Gy)<br>D35 ≤ 52Gy (35% volume ≤ 52Gy)<br>D50 ≤ 43Gy (50% volume ≤ 43Gy)<br><br>56.90Gy a´3.791Gy<br>D15 ≤ 62Gy (15% volume ≤ 62Gy)<br>D25 ≤ 53.5Gy (25% volume ≤ 53.5Gy)<br>D35 ≤ 49.2Gy (35% volume ≤ 49.2Gy)<br>D50 ≤ 40.7Gy (50% volume ≤ 40.7Gy)<br><br>36Gy a´3Gy<br>D15 ≤ 34.8Gy (15% volume ≤ 34.8Gy)<br>D25 ≤ 30Gy (25% volume ≤ 30Gy)<br>D35 ≤ 27.6Gy (35% volume ≤ 27.6Gy)<br>D50 ≤ 22.8Gy (50% volume ≤ 22.8Gy)	Vigneault et al.
Bladder neck	72.8Gy a´ 2.91Gy//63.7Gy a´3.749Gy<br>D15 ≤ 73.8Gy (15% volume ≤ 73.8Gy)<br>D25 ≤ 69.6Gy (25% volume ≤ 69.6Gy)<br>D35 ≤ 64.8Gy (35% volume ≤ 64.8Gy)<br>D50 ≤ 60Gy (50% volume ≤ 60Gy)<br><br>35.3Gy a´3.53Gy<br>D15 ≤ 39.4Gy (15% volume ≤ 39.4Gy)<br>D25 ≤ 37.1Gy (25% volume ≤ 37.1Gy)<br>D35 ≤ 34.6Gy (35% volume ≤ 34.6Gy)<br>D50 ≤ 32Gy (50% volume ≤ 32Gy)<br><br>70Gy a´2.8Gy<br>D15 ≤ 69.5Gy (15% volume ≤ 69.5Gy)<br>D25 ≤ 65.5Gy (25% volume ≤ 65.5Gy)<br>D35 ≤ 61Gy (35% volume ≤ 61Gy)<br>D50 ≤ 56.5Gy (50% volume ≤ 56.5Gy)<br><br>56.90Gy a´3.791Gy<br>D15 ≤ 65.8Gy (15% volume ≤ 65.8Gy)<br>D25 ≤ 62Gy (25% volume ≤ 62Gy)<br>D35 ≤ 57.8Gy (35% volume ≤ 57.8Gy)<br>D50 ≤ 53.5Gy (50% volume ≤ 53.5Gy)	Vigneault et al.

	<u>36Gy a´3Gy</u><br>D15 ≤ 36.9Gy (15% volume ≤ 36.9Gy)<br>D25 ≤ 34.8Gy (25% volume ≤ 34.8Gy)<br>D35 ≤ 32.4Gy (35% volume ≤ 32.4Gy)<br>D50 ≤ 30Gy (50% volume ≤ 50Gy)	
Urethra	*D 0.2% < 125%*	Murray et al.

Supplemental Table S3. Target and organs at risk dose constraints for normofractionated VMAT > 80Gy

Structure	Optimization parameter	Literature
Planning target volume (PTV)	PTV minimum ≥ 95%<br>PTV maximum ≤ 107%	
Anterior rectal wall	D15 ≤ 75Gy (15% volume ≤ 75Gy)<br>D25 ≤ 65Gy (25% volume ≤ 65Gy)<br>D35 ≤ 60Gy (35% volume ≤ 60Gy)<br>D50 ≤ 50Gy (50% volume ≤ 50Gy)	Vigneault et al.
Bladder neck	D15 ≤ 80Gy (15% volume ≤ 80Gy)<br>D25 ≤ 75Gy (25% volume ≤ 75Gy)<br>D35 ≤ 65Gy (35% volume ≤ 65Gy)<br>D50 ≤ 55Gy (50% volume ≤ 55Gy)	Vigneault et al.
Urethra	*D 0.2% < 125%*	Murray et al.

Supplemental Table S4. Target and organs at risk dose constraints for moderately hypofractionated VMAT

Structure	Optimization parameter	Literature
Planning target volume (PTV)	*PTV minimum* ≥ 95%<br>*PTV maximum* ≤ 107%	
Anterior rectal wall	*60Gy a`3Gy*<br>*D15 ≤ 58Gy (15% volume ≤ 58Gy)*<br>*D25 ≤ 50Gy (25% volume ≤ 50Gy)*<br>*D35 ≤ 46Gy (35% volume ≤ 46Gy)*<br>*D50 ≤ 38Gy (50% volume ≤ 38Gy)*<br><br>*66Gy a`3Gy*<br>D15 ≤ 63.8Gy (15% volume ≤ 63.8Gy)<br>D25 ≤ 55Gy (25% volume ≤ 55Gy)<br>D35 ≤ 50.6Gy (35% volume ≤ 50.6Gy)<br>D50 ≤ 41.8Gy (50% volume ≤ 41.8Gy)<br><br>*37.5Gy a 2.5Gy*<br>*D15 ≤ 36Gy (15% volume ≤ 36Gy)*<br>*D25 ≤ 31Gy (25% volume ≤ 31Gy)*<br>*D35 ≤ 29Gy (35% volume ≤ 29Gy)*<br>*D50 ≤ 24Gy (50% volume ≤ 24Gy)*<br><br>84 Gy a 3 Gy<br>*D15 ≤ 81.2Gy (15% volume ≤ 81.2Gy)*<br>*D25 ≤ 70Gy (25% volume ≤ 70Gy)*<br>*D35 ≤ 64.4Gy (35% volume ≤ 64.4Gy)*<br>*D50 ≤ 53.2Gy (50% volume ≤ 53.2Gy)*	*Vigneault et al.*
Bladder neck	*60Gy a`3Gy*<br>*D15 ≤ 61.5Gy (15% volume ≤ 61.5Gy)*<br>*D25 ≤ 58Gy (25% volume ≤ 58Gy)*<br>*D35 ≤ 54Gy (35% volume ≤ 54Gy)*<br>*D50 ≤ 50Gy (50% volume ≤ 50Gy)*<br><br>*66Gy a`3Gy*<br>D15 ≤ 67.6Gy (15% volume ≤ 67.6Gy)<br>D25 ≤ 63.8Gy (25% volume ≤ 63.8Gy)<br>D35 ≤ 59.4Gy (35% volume ≤ 59.4Gy)<br>D50 ≤ 55Gy (50% volume ≤ 50Gy)<br><br>*37.5Gy a 2.5Gy*<br>*D15 ≤ 38Gy (15% volume ≤ 38Gy)*<br>*D25 ≤ 36Gy (25% volume ≤ 36Gy)*<br>*D35 ≤ 34Gy (35% volume ≤ 34Gy)*<br>*D50 ≤ 31Gy (50% volume ≤ 31Gy)*<br><br>84 Gy a 3 Gy<br>*D15 ≤ 86.1Gy (15% volume ≤ 86.1Gy)*<br>*D25 ≤ 81.2Gy (25% volume ≤ 81.2Gy)*<br>*D35 ≤ 75.6Gy (35% volume ≤ 75.6Gy)*<br>*D50 ≤ 70Gy (50% volume ≤ 70Gy)*	*Vigneault et al.*
Urethra	*D0.2 < 125%*	*Murray et al.*

Supplemental Table S5. Target and organs at risk dose constraints for normofractionated VMAT 46Gy

Structure	Optimization parameter	Literature
Planning target volume (PTV)	*PTV minimum* $\geq$ 95%<br>*PTV maximum* $\leq$ 107%	
Anterior rectal wall	*D15 ≤ 44Gy (15% volume ≤ 44Gy)*<br>*D25 ≤ 38Gy (25% volume ≤ 38Gy)*<br>*D35 ≤ 20Gy (35% volume ≤ 20Gy)*<br>*D50 ≤ 29Gy (50% volume ≤ 29Gy)*	*Vigneault et al.*
Bladder neck	*D15 ≤ 47Gy (15% volume ≤ 47Gy)*<br>*D25 ≤ 44Gy (25% volume ≤ 44Gy)*<br>*D35 ≤ 38Gy (35% volume ≤ 38Gy)*<br>*D50 ≤ 32Gy (50% volume ≤ 32Gy)*	*Vigneault et al.*
Urethra	*D0.2 < 125%*	*Murray et al.*

Supplemental Table S6. Target and organs at risk dose constraints for ultra- hypofractionated VMAT

Structure	Optimization parameter	Literature
Planning target volume (PTV)	*V35Gy >95%*<br>*V37.5Gy >95%*	*Chatzikonstantinou et al.*
Anterior rectal wall	*Dmax < 38Gy*<br>*V36Gy < 1cm3 and <5%*<br>*V29Gy < 15cm3 and <20%*<br>*V18Gy < 25cm3 and <50%*	*Chatzikonstantinou et al.*
Bladder neck	*Dmax < 38Gy*<br>*V36Gy < 10cm3 and <10%*<br>*V18Gy < 40%*	*Chatzikonstantinou et al.*
Urethra	*Dmax < 44Gy*<br>*V44Gy <20%*	*Chatzikonstantinou et al.*

Supplemental Table S7. Target and organs at risk dose constraints for HDR-BT

Structure	Optimization parameter	Literature
Planning target volume (PTV)	D90% ≥ 100%<br>V150% < 35%<br>V200% <15% (- 35%)	*Henry et al. (51)*
Anterior rectal wall	D 0.1cc ≤ 75% Total dose<br>V75% < 1cm3	*Henry et al. (51)*
Bladder neck	D0.1cc < 115% Total dose<br>V75% < 1cm3	*Henry et al. (51)*
Urethra	D0.1cc < 120% Total dose<br>V 120% < 20%	*Henry et al. (51)*

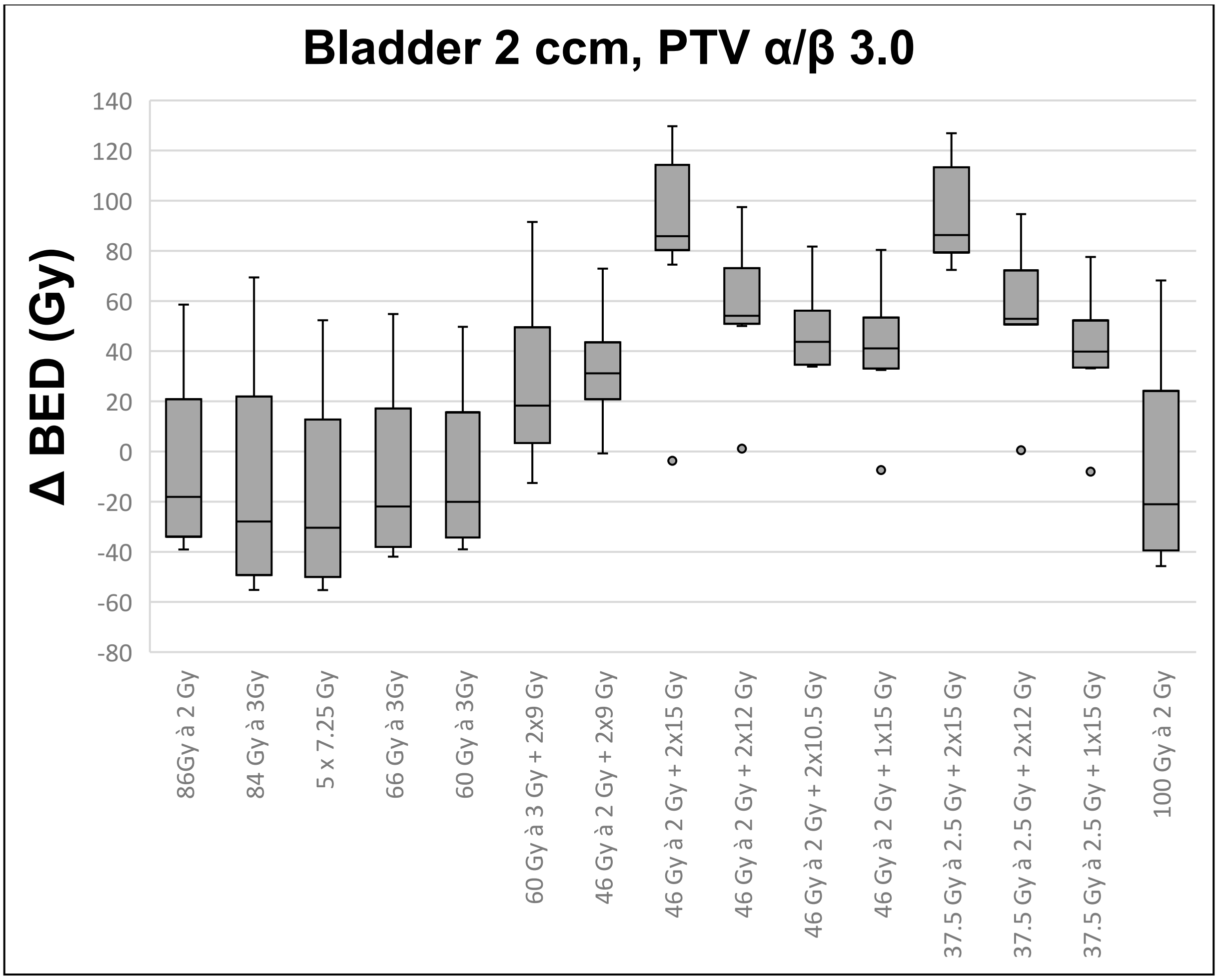

Supplemental Figure S1.Comparison of various fractionation schemes for the difference in biologically effective dose (Δ BED) between that for the target prostate volume (α/β of 3.0 assumed) and that for the highest exposed 2 ccm of the bladder (α/β of 2.0).

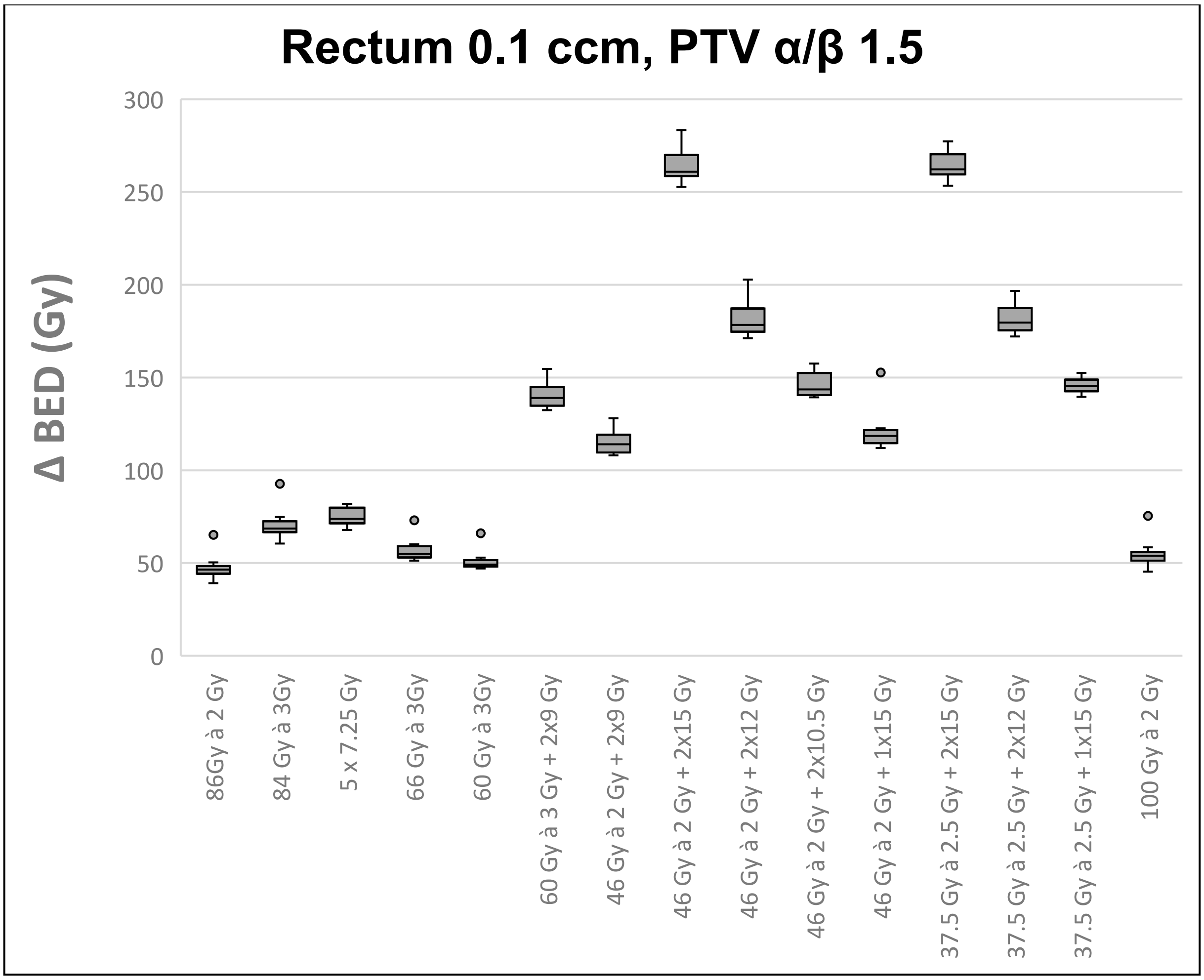

Supplemental Figure S2.Comparison of various fractionation schemes for the difference in biologically effective dose (Δ BED) between that for the target prostate volume (α/β of 1.5 assumed) and that for the highest exposed 0.1 ccm of the rectum (α/β of 3.0).
